# Aspiration as an Initial Symptom of Early-Stage Esophageal Cancer: A Case Report

**DOI:** 10.7759/cureus.69758

**Published:** 2024-09-19

**Authors:** Ryuichi Ohta, Chiaki Sano

**Affiliations:** 1 Community Care, Unnan City Hospital, Unnan, JPN; 2 Community Medicine Management, Shimane University Faculty of Medicine, Izumo, JPN

**Keywords:** adenocarcinoma, aspiration, early diagnosis, esophageal neoplasm, esophagus, family medicine, general medicine, rural, upper gastrointestinal tract

## Abstract

This case report discusses a rare presentation of esophageal adenocarcinoma located in the upper part of the esophagus in a 74-year-old male, where the sole initial symptom was silent aspiration. The absence of typical symptoms such as dysphagia or respiratory issues delayed the diagnosis of esophageal cancer. However, subsequent investigations, including endoscopy, revealed adenocarcinoma in the upper esophagus. This case underscores the importance of considering esophageal cancer in differential diagnoses when unexplained aspiration occurs, even in the absence of common symptoms, and highlights the critical need for early detection to improve patient outcomes.

## Introduction

The incidence of esophageal cancer has been steadily increasing, particularly among older patients [1]. Despite advancements in medical imaging and screening techniques, esophageal cancer is often detected at advanced stages, mainly due to the subtlety and late onset of critical symptoms [2]. One of the most concerning symptoms of esophageal cancer is aspiration. This condition significantly impairs patients' quality of life and typically emerges in the later stages of the disease [3].

However, it is essential to recognize that esophageal cancer, even in its early stages, can cause various symptoms due to the anatomical and pathophysiological relationships between the esophagus and surrounding structures [4]. Although less dramatic, these symptoms can still serve as early warning signs if adequately recognized [5].

In this report, we present a unique case of early-stage esophageal cancer where the patient’s chief complaint was recurrent aspiration during meals, occurring several times a year. Notably, this symptom was present without any of the more typical alarming signs of esophageal malignancy, such as dysphagia or significant weight loss [6]. Through this case report of the early symptoms associated with esophageal cancer, we aim to discuss general physicians' diagnostic challenges and propose earlier detection and intervention strategies. This case underscores the need for increased awareness and consideration of less common presentations of esophageal cancer in routine clinical practice.

## Case presentation

A 74-year-old man came to a rural community hospital with a chief complaint of several episodes of aspiration and choking while eating over the past year. Two years before the visit, the patient had one episode of aspiration and choking while eating dinner with his wife, causing a forceful cough that was able to clear the bolus. Similar episodes happened one year before and one month before the visit. He was anxious about pharyngeal and laryngeal cancers and came to our hospital for the investigation. He did not have the other symptoms of suspected cancer, such as chronic cough, appetite loss, body weight loss, fatigue, night sweats, pain in the throat, chest, and abdomen, and joint pain. He was an ex-smoker and smoked 10 cigarettes per day 20 years ago and drank one beer several times weekly. The past medical history included hypertension and dyslipidemia. His medications were amlodipine 5 mg daily and atorvastatin 10 mg daily.

The vital signs at the visit were as follows: blood pressure, 145/87 mmHg; pulse rate, 76 beats/min; body temperature, 36.4 °C; respiratory rate, 14 breaths/min; and oxygen saturation, 97% on room air. The patient was alert to time, place, and person. Physical examination showed no abnormalities in the mouth, throat, and neck, with no cervical lymphadenopathy. No other abnormal neurological findings were noted. There were no apparent abnormalities in the chest or abdomen and no skin eruptions. The laboratory tests showed no anemia without high inflammatory conditions (Table 1).

**Table 1:** Initial laboratory data of the patient eGFR, estimated glomerular filtration rate; CK, creatine kinase; CRP, C-reactive protein

Parameter	Level	Reference
White blood cells	6.10	3.5–9.1 × 10^3^/μL
Hemoglobin	14.6	11.3–15.2 g/dL
Hematocrit	44.7	33.4–44.9%
Mean corpuscular volume	97.0	79.0–100.0 fl
Platelets	21.4	13.0–36.9 × 10^4^/μL
Erythrocyte sedimentation rate	14	2–10 mm/hour
Total protein	7.4	6.5–8.3 g/dL
Albumin	4.8	3.8–5.3 g/dL
Total bilirubin	0.7	0.2–1.2 mg/dL
Aspartate aminotransferase	15	8–38 IU/L
Alanine aminotransferase	19	4–43 IU/L
Alkaline phosphatase	36	106–322 U/L
γ-Glutamyl transpeptidase	23	<48 IU/L
Lactate dehydrogenase	134	121–245 U/L
Blood urea nitrogen	16.9	8–20 mg/dL
Creatinine	0.86	0.40–1.10 mg/dL
eGFR	66.0	>60.0 mL/min/1.73m^2^
Serum Na	140	135–150 mEq/L
Serum K	4.0	3.5–5.3 mEq/L
Serum Cl	103	98–110 mEq/L
Serum Ca	9.7	8.8–10.2 mg/dL
Serum P	3.2	2.7–4.6 mg/dL
Serum Mg	2.0	1.8–2.3 mg/dL
CK	143	56–244 U/L
CRP	0.05	<0.30 mg/dL
Anti-nuclear antibody	40	<40
Urine test	-	-
Leukocyte	Negative	Negative
Nitrite	Negative	Negative
Protein	Negative	Negative
Glucose	Negative	Negative
Urobilinogen	Normal	Normal
Bilirubin	Negative	Negative
Ketone	Negative	Negative
Blood	Negative	Negative
pH	6.5	-
Specific gravity	1.013	-

Chest X-ray, brain computed tomography, and magnetic resonance imaging showed no abnormalities.

**Figure 1:**
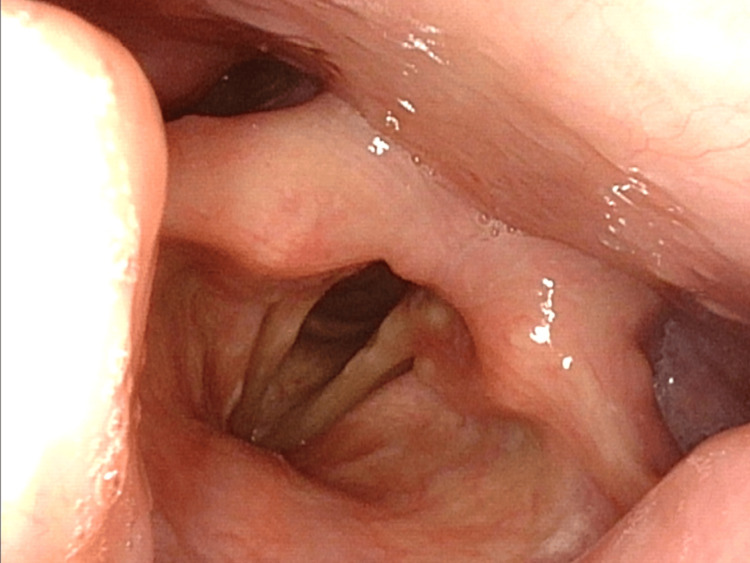
Laryngoscopy showing no abnormal lesions without dysfunction of vocal and epiglottis movements

Initially, we suspected pharyngeal and laryngeal cancers and investigated the lesions with laryngoscopy, which showed no abnormal lesions without dysfunction of vocal and epiglottis movements (Figure 1).

Neck and chest computed tomography did not show any swelling on the trachea and esophagus walls. After discussing this with the patient, we learned that he had been investigated by barium contrast examination, not upper gastrointestinal endoscopy, for cancer screening previously. Although the possibility of esophageal cancer was low, we performed an upper gastrointestinal endoscopy to investigate esophageal lesions triggering his aspiration.

**Figure 2:**
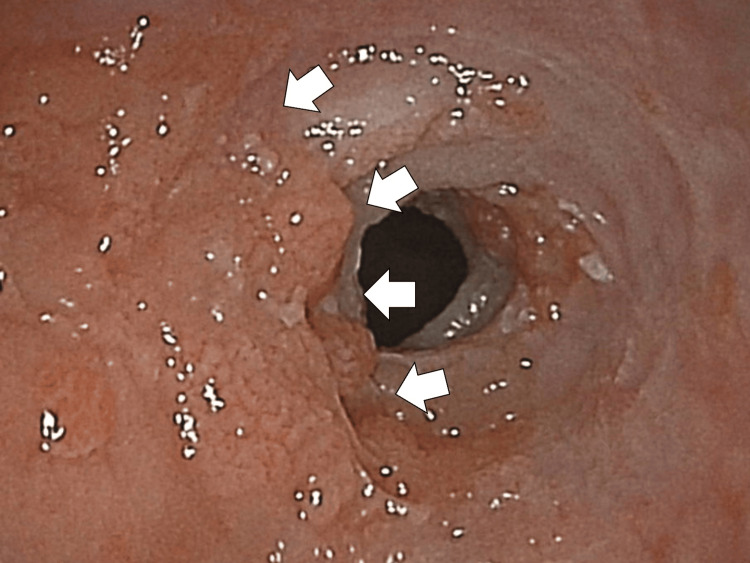
The upper gastrointestinal endoscopy clarifying the focal mass lesion with multiple ulcers in the upper part of the esophagus (white arrows)

Upper gastrointestinal endoscopy revealed the focal mass lesion with multiple ulcers, 17 cm away from the incisors, in the upper part of the esophagus (Figure 2).

**Figure 3:**
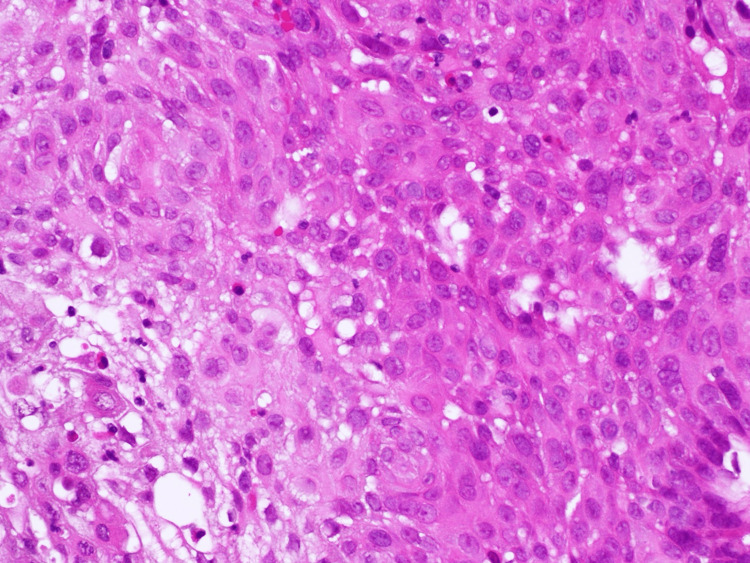
The pathological finding showing adenocarcinoma of the esophagus (hematoxylin and eosin stain, original magnification 400×)

The pathological finding showed adenocarcinoma of the esophagus (Figure 3).

As there was no lymphadenopathy, he was referred to a surgeon with the pathological grade of IA and treated by esophagectomy with esophageal reconstruction. His clinical course was favorable, and the patient is being followed as an outpatient. After the treatment, he did not experience any aspiration episodes.

Written informed consent was obtained from the patient to publish this case report and any accompanying images/information.

## Discussion

This case report highlights an uncommon but critical presentation of esophageal cancer where aspiration was the initial symptom. Aspiration as a primary complaint in esophageal cancer is rare, with most cases typically presenting with more advanced symptoms such as dysphagia, weight loss, and chest pain [7]. However, this case underscores the importance of considering esophageal cancer in the differential diagnosis when patients, especially older adults, present with recurrent aspiration without other apparent causes.

Aspiration occurs due to the failure of protective reflexes, often associated with neurological or muscular disorders [8]. In the context of esophageal cancer, the pathophysiology can be linked to the direct mechanical obstruction or impairment of the esophagus and surrounding structures, including the upper esophageal sphincter, which can lead to the misdirection of food into the airway [9]. Previous studies have documented that esophageal tumors, even in early stages, can disrupt the coordination of swallowing, leading to episodes of aspiration [10]. As observed in this case, early-stage esophageal cancer can impinge on the sensory and muscular functions of the esophageal wall and trigger disorganized constrictions of sphincter muscles, regurgitating the food. Thus, aspiration should be included as an initial symptom of esophageal cancer so as not to miss the diagnosis.

The patient's presentation with recurrent aspiration, in the absence of other common symptoms such as dysphagia or weight loss, posed a diagnostic challenge [11]. This case illustrates the need for heightened clinical suspicion and a low threshold for endoscopic evaluation, particularly in high-risk individuals with a history of smoking or alcohol use, even if their symptoms appear non-specific [12]. Research has shown that early detection of esophageal cancer significantly improves prognosis, with early-stage cancers having better outcomes compared to those diagnosed at later stages [13]. Health maintenance and annual health checks should be enhanced to detect esophageal cancers. Family physicians are system-specific specialists who help patients monitor their health and prevent critical diseases, including cancers [14]. In this case, general physicians can check previous annual health checks of patients with atypical symptoms and investigate their missed parts from various perspectives [15].

Furthermore, the absence of typical symptoms such as dysphagia in this case may be due to the tumor's location and the relatively early stage of the disease. The upper esophageal involvement, as seen in this patient, may present differently than more distal tumors, potentially leading to less obstructive symptoms and more subtle presentations like aspiration [16]. This case also emphasizes the role of systematic evaluation in primary care settings. General physicians should be aware of the varied presentations of esophageal cancer, including atypical symptoms like recurrent aspiration [17]. Early referral for endoscopy in cases of unexplained aspiration could lead to earlier diagnosis and improved patient outcomes [18]. Moreover, this case supports the need for routine cancer screening, particularly in older adults who have not previously undergone such evaluations.

## Conclusions

While aspiration is commonly associated with neurological or upper airway conditions, this case demonstrates its potential as an early indicator of esophageal cancer. Clinicians should maintain a broad differential diagnosis when evaluating patients with recurrent aspiration, particularly when other common causes have been ruled out. Early endoscopic investigation is warranted to exclude esophageal malignancy and potentially improve the patient's prognosis through timely intervention.
